# The Prostacyclin Analogue, Treprostinil, Used in the Treatment of Pulmonary Arterial Hypertension, is a Potent Antagonist of TREK-1 and TREK-2 Potassium Channels

**DOI:** 10.3389/fphar.2021.705421

**Published:** 2021-06-29

**Authors:** Kevin P. Cunningham, Lucie H. Clapp, Alistair Mathie, Emma L. Veale

**Affiliations:** ^1^ Medway School of Pharmacy, University of Kent and University of Greenwich, Chatham Maritime, United Kingdom; ^2^ Wolfson Centre for Age-Related Diseases, King’s College London, London, United Kingdom; ^3^ Institute of Cardiovascular Science, University College London, London, United Kingdom; ^4^ School of Engineering, Arts, Science and Technology, University of Suffolk, Ipswich, United Kingdom

**Keywords:** treprostinil (PubChem CID: 6918140), pulmonary arterial hypertension, TREK-1 (tandem of pore domain in a weak inwardly rectifying K channel (Twik)-related K channels), TASK-1 channel, BL-1249, TREK-2 channels, K2P channels

## Abstract

Pulmonary arterial hypertension (PAH) is an aggressive vascular remodeling disease that carries a high morbidity and mortality rate. Treprostinil (Remodulin) is a stable prostacyclin analogue with potent vasodilatory and anti-proliferative activity, approved by the FDA and WHO as a treatment for PAH. A limitation of this therapy is the severe subcutaneous site pain and other forms of pain experienced by some patients, which can lead to significant non-compliance. TWIK-related potassium channels (TREK-1 and TREK-2) are highly expressed in sensory neurons, where they play a role in regulating sensory neuron excitability. Downregulation, inhibition or mutation of these channels leads to enhanced pain sensitivity. Using whole-cell patch-clamp electrophysiological recordings, we show, for the first time, that treprostinil is a potent antagonist of human TREK-1 and TREK-2 channels but not of TASK-1 channels. An increase in TASK-1 channel current was observed with prolonged incubation, consistent with its therapeutic role in PAH. To investigate treprostinil-induced inhibition of TREK, site-directed mutagenesis of a number of amino acids, identified as important for the action of other regulatory compounds, was carried out. We found that a gain of function mutation of TREK-1 (Y284A) attenuated treprostinil inhibition, while a selective activator of TREK channels, BL-1249, overcame the inhibitory effect of treprostinil. Our data suggests that subcutaneous site pain experienced during treprostinil therapy may result from inhibition of TREK channels near the injection site and that pre-activation of these channels prior to treatment has the potential to alleviate this nociceptive activity.

## Introduction

Pulmonary arterial hypertension (PAH) is a progressive vascular remodeling disease which eventually leads to right ventricular heart failure and premature death (Simonneau et al., 2019). A rare disease, it has an estimated adult incidence of 5.8 per million, with a higher incidence noted in females and a mean patient age between 50–65 years (Galie et al., 2015; Leber et al., 2021). There have been a number of advances in treatments, which have transformed the quality of life of patients and reduced mortality. However, despite these advances the prognosis for sufferers remains poor with a 5-years survival rate of 61–65% (Farber et al., 2015). It is widely surmised that PAH is initiated following damage to the endothelium (Clapp and Gurung, 2015; Madonna et al., 2015). Endothelial dysfunction leads to a decrease in the production of the vasodilators prostacyclin (prostaglandin I_2_; PGI_2_) and nitric oxide (NO) and an increase in the potent vasoconstrictors, endothelin (ET-1) and thromboxane, which combine to increase vascular tone, cell proliferation and platelet aggregation in pulmonary arteries, to drive narrowing of blood vessels (Morrell et al., 2009; Hemnes and Humbert, 2017). Given PGI_2_ is a key vasoactive regulator released by endothelial cells in the pulmonary arteries, initial, therapeutic management of PAH involved giving epoprostenol (synthetic PGI_2_), but due to its short chemical and biological half-life led to the development of PGI_2_ stable analogues, iloprost, beraprost and treprostinil or more recently the non-prostanoid PGI_2_ receptor agonist, selexipag. These therapies improve exercise capacity, breathing, hemodynamic circulation, and patient survival in various trials (Gomberg-Maitland and Olschewski, 2008; Sitbon and Vonk Noordegraaf, 2017).

Treprostinil is a tricyclic benzindene analogue of PGI_2_ which can be administered orally, subcutaneously (SC), intravenously or inhaled (Lindegaard Pederson et al., 2020), with SC administration (Remodulin) approved by the US Food and Drug Administration and the World Health Organization. In a number of studies, SC treprostinil has demonstrated significant improvements in PAH symptoms and a reduction in adverse events compared to other delivery mechanisms and other synthetic PGI_2_ analogues (Picken et al., 2019). A potent vasodilator, its therapeutic effects occur via activation of specific prostanoid receptors (Clapp and Gurung, 2015; Corboz et al., 2021). One major limitation of SC treprostinil treatment is that severe site pain is the main significant adverse event experienced by patients (Picken et al., 2019). The origin of this site pain is unknown but is likely to involve the regulation of ion channel activity in nociceptive sensory neurons.

The TWIK-related (TREK-1 and TREK-2) potassium channels are members of the two-pore domain (K_2P_) family of ion channels. TREK-1 and TREK-2 have been shown to be highly expressed in sensory neurons, mainly in small nociceptors dorsal root ganglion (DRG), with TREK-2 expression identified in IB4-binding C-fibre nociceptors and TREK-1 expression in astrocytes and neurons of the spinal cord, where they have been shown to regulate spontaneous pain, neuropathic pain and hyperalgesia (Alloui et al., 2006; Kang and Kim, 2006; Acosta et al., 2014; Pereira et al., 2014; Viatchenko-Karpinski et al., 2018). Moreover, TREK-1 and TREK-2 knockout (KO) mice show increased sensitivity to a range of noxious stimuli, including heat, mechanical and inflammatory agents (Alloui et al., 2006; Noel et al., 2009; Pereira et al., 2014). Conversely, TREK channel activation by riluzole, BL-1249, GI-530159, RNE28 and C3001a was found, respectively, to eliminate oxaliplatin-induced pain (Poupon et al., 2018), decrease tactile allodynia in neuropathic rats (Garcia et al., 2020), reduce excitability of small DRG neurons (Loucif et al., 2018), increase antinociceptive activity in mouse models of pain (Busserolles et al., 2020) and alleviate stimuli-induced hyperalgesia, allodynia and inflammation in mice (Qiu et al., 2020).

The aim of this study was to determine whether treprostinil had a direct inhibitory action on human cloned TREK-1 and TREK-2 channels and thus provide a plausible explanation for the severe site-pain experienced by PAH patients treated with this drug. We investigated treprostinil effects on a number of TREK channels with point mutations of amino acids required for the modulatory action of a number of other known TREK channel inhibitors and activators. Finally, using a selective activator of TREK channels, we investigated whether we could overcome the inhibitory effect of treprostinil on these channels. A preliminary account of some of these data has been reported previously (Cunningham et al., 2018).

## Materials and Methods

Most of the methods used here have been described previously (Veale et al., 2014; Cunningham et al., 2019; Mathie et al., 2021a) and will only be mentioned in brief below.

### Mammalian Expression Plasmids

TREK-1 (KCNK2, Genbank™ NP_055032.1) and TASK-1 (KCNK3, NP_002237) cDNA were cloned into the pcDNA3.1^+^ vector (Invitrogen, Carlsbad, CA, United States) and TREK-2 (KCNK10, NP_612190.1) into PCMV6-XL-4 (OriGene Technologies, Inc. United States).

### Mutagenesis

Ligand binding site and gating mutations were introduced by site-directed mutagenesis into human TREK-1 (Q76A, I80A, L102A, Y270A, Y284A, L289A) and TREK-2 (L320A, K302Q) cDNA using the QuikChange kit (Agilent, CA, United States) as previously described (Veale et al., 2014). DNA sequencing was performed by DNA Sequencing and Services (MRC/PPU, School of Life Sciences, University of Dundee, Scotland).

### Cell Culture

All experiments were performed using a modified human embryonic kidney 293 cell line, tsA201 (European Collection of Authenticated Cell Cultures; Sigma-Aldrich, United Kingdom), prepared and maintained as previously described (Veale et al., 2014). Cells were split when at a confluency of 80%, resuspended in media to a concentration of 7 × 10^4^ and then 0.5 ml plated into a four-well plate containing 13 mm poly-d-lysine coated (1 mg ml^−1^) glass coverslips, ready for transfection the following day.

### Transfection

Plasmids containing cDNA for either wildtype (WT) or mutated TREK cDNA and a similar plasmid encoding the cDNA for green fluorescent protein (GFP), were co-transfected at a concentration of 0.5 µg using a modified calcium-phosphate protocol, as previously described (Cunningham et al., 2019). All mutant channels were expressed as homodimeric channels, where each α-subunit expressed the incorporated mutation.

### Whole-Cell Patch-Clamp Electrophysiology

Currents were recorded from GFP fluorescing tsA201 cells expressing the cDNA of interest using whole-cell patch-clamp in a voltage-clamp configuration and a step-ramp voltage protocol using an extracellular solution composed of 145 mM NaCl, 2.5 mM KCl, 3 mM MgCl_2_, 1 mM CaCl_2_ and 10 mM HEPES (pH adjusted to 7.4 with NaOH) and an intracellular pipette solution of 150 mM KCl, 3 mM MgCl_2_, 5 mM EGTA and 10 mM HEPES (pH adjusted to 7.4 with KOH). All experiments were conducted at room temperature (20–25°C) and currents were recorded using an Axopatch 1D patch clamp amplifier (Molecular Devices, Sunnyvale, CA), filtered at 2 kHz and digitized at 5 kHz. Control solution and modulatory compounds were perfused at a rate of 4–5 ml min^−1^ for ∼3–6 min per experiment. For incubation studies, the cells were incubated in either extracellular solution or extracellular solution containing desired concentration of treprostinil for 20 min, prior to commencing electrophysiological recording and for the duration of the experiment.

### Data Analysis and Statistics

Data analysis of whole-cell outward current and analysis software was as previously described in Cunningham et al. (2019). Whole-cell outward currents were measured in picoamps (pA) and recorded as the difference current between that measured at −40 and −80 mV, normalized against cell capacitance (pF). Current-voltage graphs were obtained from the voltage ramp (−120 mV to +20 mV). All raw data traces were averages of all cells recorded in each condition. Data were expressed as the mean ±95% Confidence Intervals (CI), and *n* represents the number of individual cells, displayed as symbols on the graphs. Statistical analysis used were either a one-way ANOVA with a post-hoc Dunnett’s multiple comparisons test or a paired Student’s *t*-test. Data was considered statistically different if *p* < 0.05 (*), *p* < 0.01 (**), *p* < 0.001 (***). Data from cells expressing mutant channels were compared with matched control data from either WT TREK-1 or WT TREK-2 recorded either simultaneously or around the same calendar period and cell batch number.

### Chemicals

BL-1249 was purchased from Sigma-Aldrich, United Kingdom and dissolved in dimethyl sulfoxide (DMSO) to create a 10 mM stock solution. Treprostinil (CAY10162) was purchased from Cambridge Bioscience, United Kingdom (distributor for Cayman Chemical Co.) and dissolved in DMSO to a concentration of 10 mM. Dilutions of the stock solutions were made directly into the extracellular solution for use the same day.

## Results

### TREK-1 and TREK-2 Channels are Potently Inhibited by Treprostinil

**FIGURE 1:**
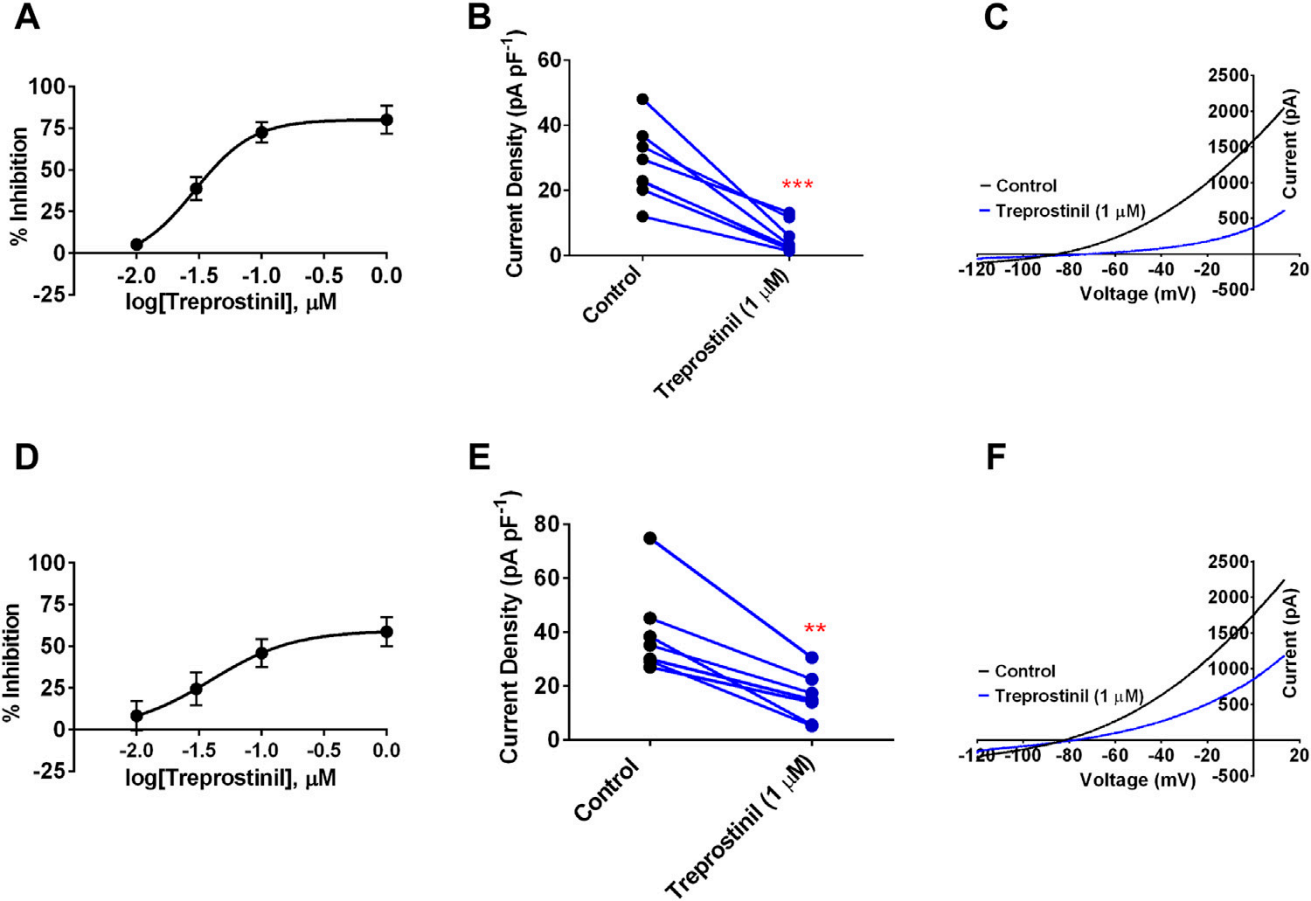
Effect of treprostinil on human cloned TREK-1 and TREK-2 channels **(A)** Concentration-response curve for treprostinil inhibition of human TREK-1 current. Error bars represent standard error of the mean (SEM) **(B)** Measurement of whole-cell TREK-1 current (pA) normalized against cell capacitance (pF) in control 2.5 mM [K^+^] solution (black symbols) and following acute application of treprostinil (1 μM, blue symbols, ***p < 0.0002 (95% CI: −31.4 to −14.5), paired *t*-test **(C)** Current-voltage plot of TREK-1 currents (average of *n* = 8 cells) under control conditions (black line) and in the presence of treprostinil (1 μM, average of *n* = 8 cells, blue line) recorded over a voltage ramp (−120 mV to +20 mV) **(D)** Concentration-response curve for treprostinil inhibition of human TREK-2 current **(E)** Measurement of whole-cell TREK-2 current (pA pF^−1^) in control and following acute application of treprostinil (1 μM, **p < 0.001 [95% CI: −34.43 to −14.18]), paired *t*-test **(F)** Current-voltage plot of TREK-2 in control (black line, *n* = 7) and in the presence of treprostinil (1 μM, *n* = 7, blue line).

We first investigated whether TREK-1 and TREK-2 channel current was directly affected by PGI_2_ stable analogue, treprostinil. Application of treprostinil over a concentration range of 0.01–1 µM to cells expressing WT human TREK-1 channels resulted in a potent inhibition of whole-cell outward current that gave a calculated 50% inhibitory concentration (IC_50_) of 0.03 µM [95% confidence Intervals (CI): 0.01 to 0.06] estimated from the difference between current measured at −40 mV and −80 mV (Figure 1A). Using a maximal concentration of treprostinil (1 µM), we observed a potent inhibition of whole-cell outward current from 28.2 pA pF^−1^ [95% CI: 18.8 to 37.6, *n* = 8] in control to 5.3 pA pF^−1^ (95% CI: 1.4 to 9.2, *n* = 8) when treprostinil was present (Figures 1B,C). Similarly, application of treprostinil over a concentration range of 0.01–1 µM to cells expressing WT TREK-2 channels, resulted in a calculated IC_50_ of 0.04 µM (95% CI: 0.004 to 0.39) (Figure 1D). Where at a concentration of 1 μM, the averaged TREK-2 current of 39.9 pA pF^−1^ (95% CI: 24.6 to 55.3, *n* = 7) in control solution was reduced to 18.7 pA pF^−1^ (95% CI: 7.3 to 24.0, *n* = 7) in the presence of treprostinil (Figures 1E,F).

### Treprostinil Does Not Regulate TASK-1 Channels Directly

**FIGURE 2:**
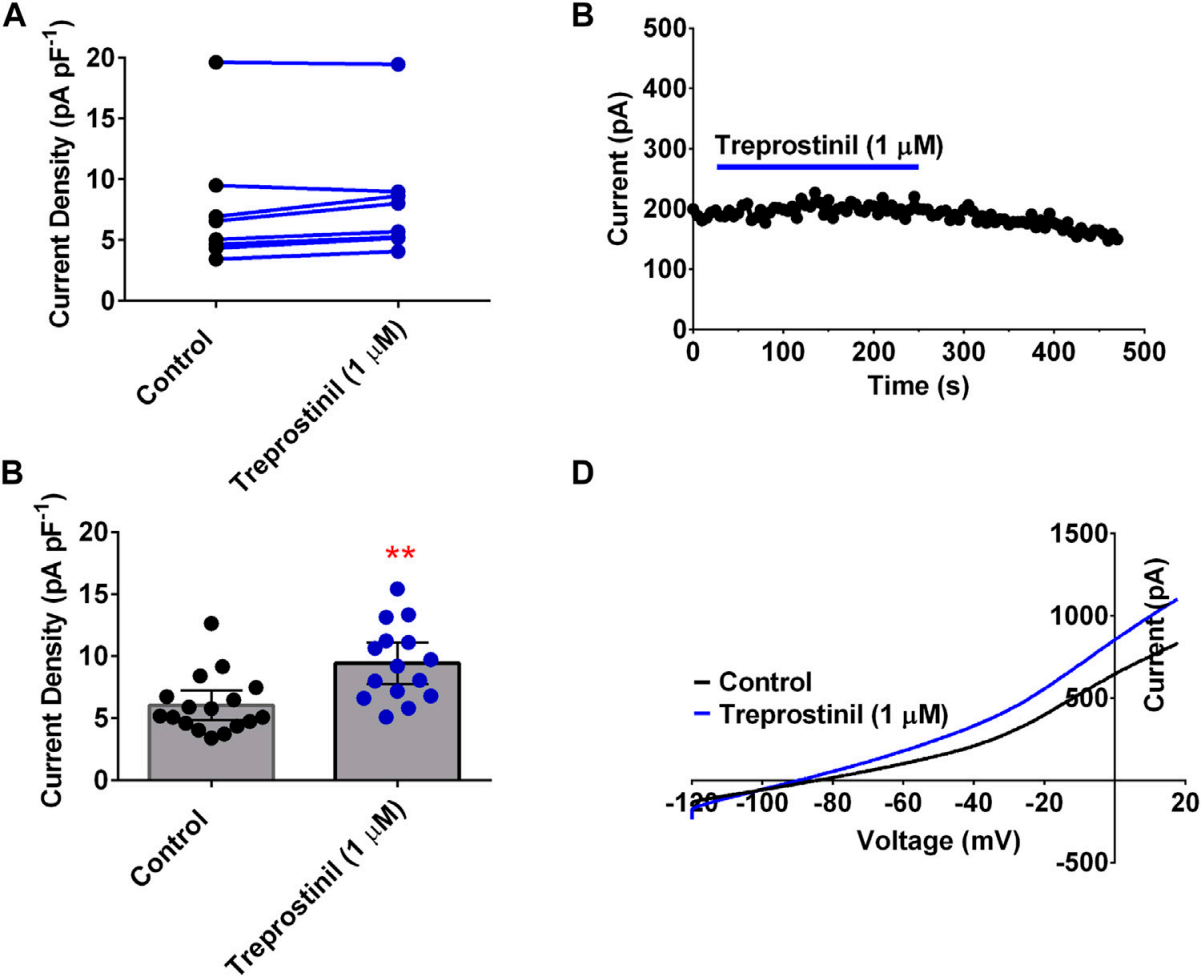
Effect of treprostinil on human TASK-1 (A) Measurement of whole-cell TASK-1 current (pA pF^−1^) in control solution (black symbols) and following application of treprostinil (1 μM, blue symbols, *p* = 0.506 [95% CI: −53.06 to 12.66]); paired *t*-test **(B)** Exemplar time course, demonstrating the effect of 1 µM treprostinil (drug application is represented by the blue line), on whole-cell TASK-1 current (pA) over time (s). Each point is a 50 s average of the difference current **(C)** Bar graphs of current densities (pA pF^−1^) from cells transiently expressing WT TASK-1 measured in control extracellular solution and when cells have been incubated in 1 µM treprostinil (>20 min), **p = 0.0013 [95%CI: 1.4 to 5.3]), unpaired *t*-test. Error bars represent the 95% CI **(D)** Current-voltage plot of TASK-1 in control conditions (black line) and treprostinil (1 μM, blue line) recorded over a voltage ramp (−120 mV to +20 mV).

To understand whether this inhibitory effect of treprostinil on the TREK channels was selective for this channel subtype, we tested it on another member of the K_2P_ family of channels, namely TASK-1, which has been widely, implicated in PAH pathogenesis (Ma et al., 2013; Boucherat et al., 2015; Antigny et al., 2016; Navas et al., 2017; Cunningham et al., 2019). Unlike for TREK-1 and TREK-2, treprostinil had neither an inhibitory nor activatory effect on WT human TASK-1 channels, using the same experimental protocol. Average current density for TASK-1 channels measured in control solution was 7.2 pA pF^−1^ (95% CI: 1.4 to 13.0, *n* = 5) compared to 6.5 pA pF^−1^ (95% CI: 3.2 to 9.8, *n* = 5) in the presence of treprostinil (1 μM; *p* > 0.05 (95% CI: −3.4 to 2.0), paired *t*-test, Figures 2A,B). As treprostinil appeared not to inhibit the TASK-1 channel directly, we sought to investigate whether treprostinil was able to exert an effect on TASK-1 via the activation of signaling pathways, such as the protein kinases (PKA)-dependent pathway, as has been previously suggested (Olschewski et al., 2006; Corboz et al., 2021). To do this we pre-incubated TASK-1 expressing tsA201 cells either in control solution (untreated) or with the addition of 1 µM treprostinil (treated) for 20 min and then measured currents in each condition. An increase (*p* < 0.05, unpaired *t*-test) in current density was observed in treprostinil-incubated cells (9.4 pA pF^−1^ [95% CI: 7.7 to 11.1, *n* = 15]), compared with untreated control-incubated cells (6.0 pA pF^−1^ [95% CI: 4.8 to 7.2, *n* = 17], Figures 2C,D), with an average current increase of 36.2%.

### Treprostinil Inhibition of TREK-1 is Not Affected by Known Allosteric Ligand-Binding Site Mutations Located in the Extracellular Cap

**FIGURE 3:**
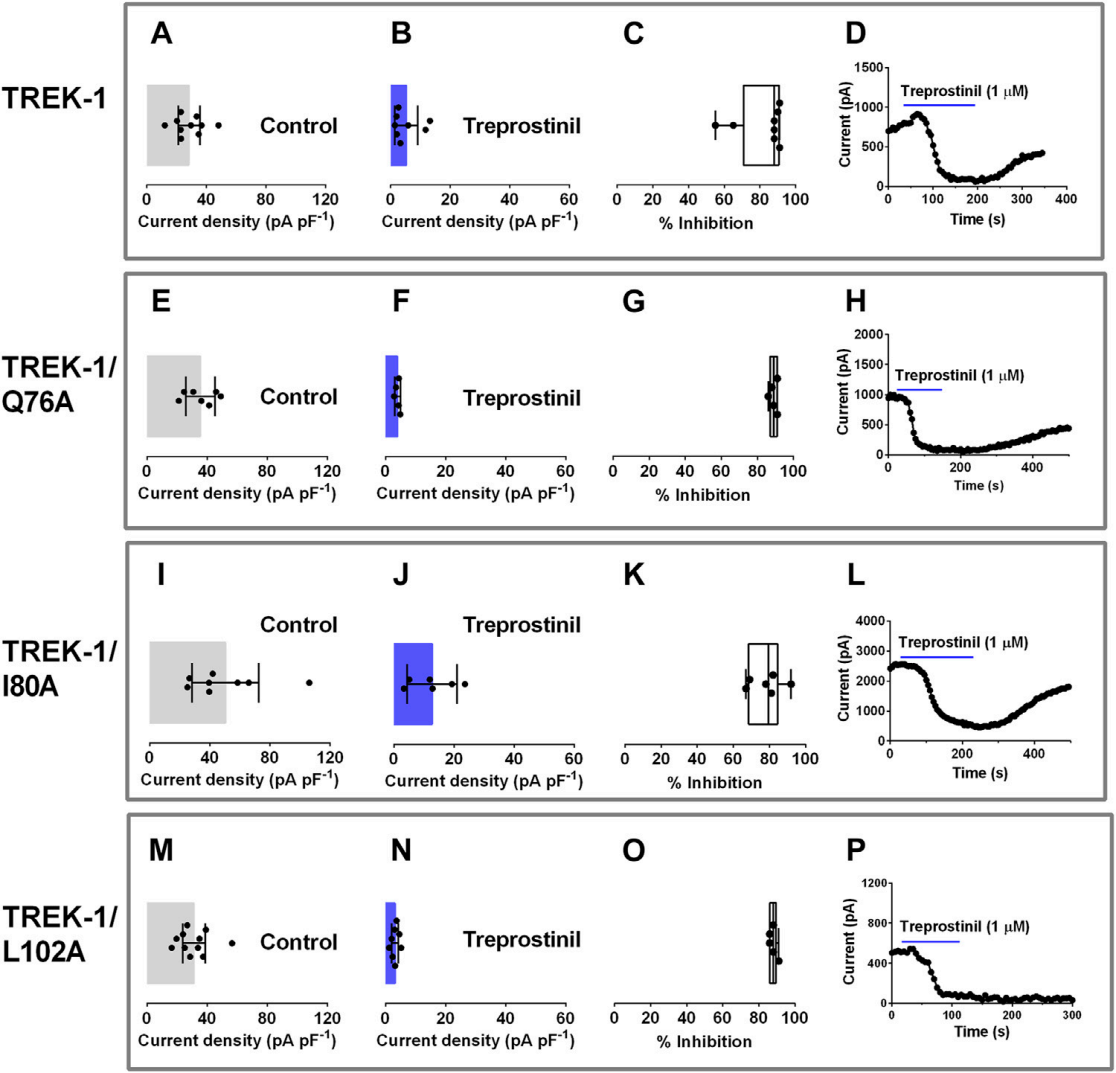
Effect of treprostinil on extracellular allosteric ligand-binding site mutations in TREK-1. Graphs of current densities (pA pF^−1^) from cells transiently expressing WT TREK-1 **(A, B)**, TREK-1/Q76A (E, F), TREK-1/I80A **(I, J)** and TREK-1/L102A **(M, N)** in control extracellular solution and in the presence of 1 µM treprostinil. Error bars represent the 95% CI and symbols represent currents measured from individual cells **(C, G, K, O)** Box and Whisker plots of treprostinil (1 µM) inhibition of WT TREK-1, TREK-1/Q76A, TREK-1/I80A and TREK-1/L102A. Bars represent the min and max inhibition and lines the median inhibition, while symbols represent the individual, data points **(D, H, L, P)** Exemplar time courses, demonstrating the effect of 1 µM treprostinil (application shown by the blue line), on whole cell current (pA), for each channel type.

We next sought to elucidate the molecular mode of action of treprostinil on the TREK channel. It has previously been shown that some inhibitors of TREK channels act by binding to sites in the extracellular cap of these channels resulting in a block of the ion conduction pathway (Luo et al., 2017). We mutated three residues, glutamine (Q) 76, isoleucine (I) 80 and leucine (L) 102 to alanine’s (A) and compared the treprostinil-induced inhibition of these mutant channels to WT TREK-1 since mutations of these sites has been shown to reduce the effectiveness of N-(4-cholorphenyl)-N-(2-(3,4-dihydrosioquinolin-2 (1H)-yl)-2-oxoethyl) methanesulfonamide (TKDC)-induced allosteric conformational transitions of the extracellular cap (Luo et al., 2017). Alanine substitution of these amino acids (Q76A, I80A and L102A) into TREK-1 resulted in functional homodimeric channels where the mutation is expressed on both α-subunits, with whole-cell current densities (pA pF^−1^) either similar to WT (Q76A and L102A) or slightly increased (I80A, *p* < 0.03, one-way ANOVA, followed by Dunnett’s multiple comparisons test, Figures 3A,E,I, M). The average whole-cell current was 28.3 pA pF^−1^ (95% CI: 21.0 to 35.7, *n* = 10) for WT; 35.6 pA pF^−1^ (95% CI: 25.8 to 45.4, *n* = 7) for Q76A; 50.4 pA pF^−1^ (95% CI: 28.2 to 72.6, *n* = 8) for I80A; 31.1 pA pF^−1^ (95% CI: 23.7 to 38.6, *n* = 11) for L102A. Ramp changes in holding potential from −120 to +20 mV showed that mutant channels were outwardly rectifying with mean zero current reversal potentials that were not significantly different (*p* > 0.05, one-way ANOVA followed by a Dunnett’s multiple comparisons test) from WT channels (−81.4 mV [95% CI: = −87.3 to -75.6, *n* = 7] for Q76A; −86.1 mV [−89.9 to −82.3, *n* = 8] for I80A; −85.6 mV [−89.7 to −81.5, *n* = 11] and −83.1 mV [95% CI: = −87.3 to −78.9, *n* = 10] for WT) and close to the expected reversal potential for a potassium selective channel under these experimental conditions.

We then investigated the influence of these mutations on the inhibitory effect produced by treprostinil on WT TREK-1 channels. Application of treprostinil (1 µM), was found to potently inhibit the current of all three mutated channels (Q76A: 4.0 pA pF^−1^ [95% CI: 3.0 to 5.0, *n* = 5]; I80A: 12.7 pA pF^−1^ [95% CI: 4.5 to 21.0, *n* = 6]; L102A: 3.2 pA pF^−1^ [95% CI: 2.1 to 4.3, *n* = 8]), similar to WT channels (5.3 pA pF^−1^ [95% CI: 1.4 to 9.2, *n* = 10] (Figures 3B,F,J,N). Overall inhibition of the current by treprostinil for each mutant channel was 89% (95% CI: 86.3 to 91.6, *n* = 5) for Q76A, 78.2% (95% CI: 68.5 to 87.8, *n* = 6) for I80A and 88% (95% CI: 85.3 to 90.3, *n* = 5) for L102A which were similar (*p* > 0.05) to WT, 82% (95% CI: 70.4 to 93.6, *n* = 8), (Figures 3C,G,K,O), represented by exemplar time courses for each condition (Figures 3D,H,L,P).

### Disruption of Known Contact Binding Sites for the Antagonist Norfluoxetine and Activators BL-1249, ML335 and ML402 Does Not Interfere With Treprostinil Inhibition of Either TREK-2 or TREK-1

The antidepressant, norfluoxetine an active metabolite of fluoxetine (Prozac) and a known inhibitory molecule of the TREK family (Kennard et al., 2005; Heurteaux et al., 2006) has been resolved bound within the intramembrane fenestrations, located below the selectivity filter of TREK-2 (Dong et al., 2015). A number of amino acids were identified as contact points for norfluoxetine, including a phenylalanine (F) at position 316 (F316) and a leucine at position 320 (L320) in the TM4 region of the channel. Furthermore, mutation of L320 was found to reduce norfluoxetine’s inhibition of TREK-2 (Dong et al., 2015). The same amino acid on the TM4 of TREK-1 has also been identified as a contact point for the activator, BL-1249 and mutation of this corresponding site, reduced the effectiveness of the compound (Schewe et al., 2019). As the inhibitory profile of treprostinil on TREK-2 and TREK-1 is similar to that observed for fluoxetine and norfluoxetine (Kennard et al., 2005; Dong et al., 2015) we looked to determine whether treprostinil was exerting its inhibitory effect via the equivalent amino acids on the intramembrane fenestrations of both TREK-2 and TREK-1.

**FIGURE 4:**
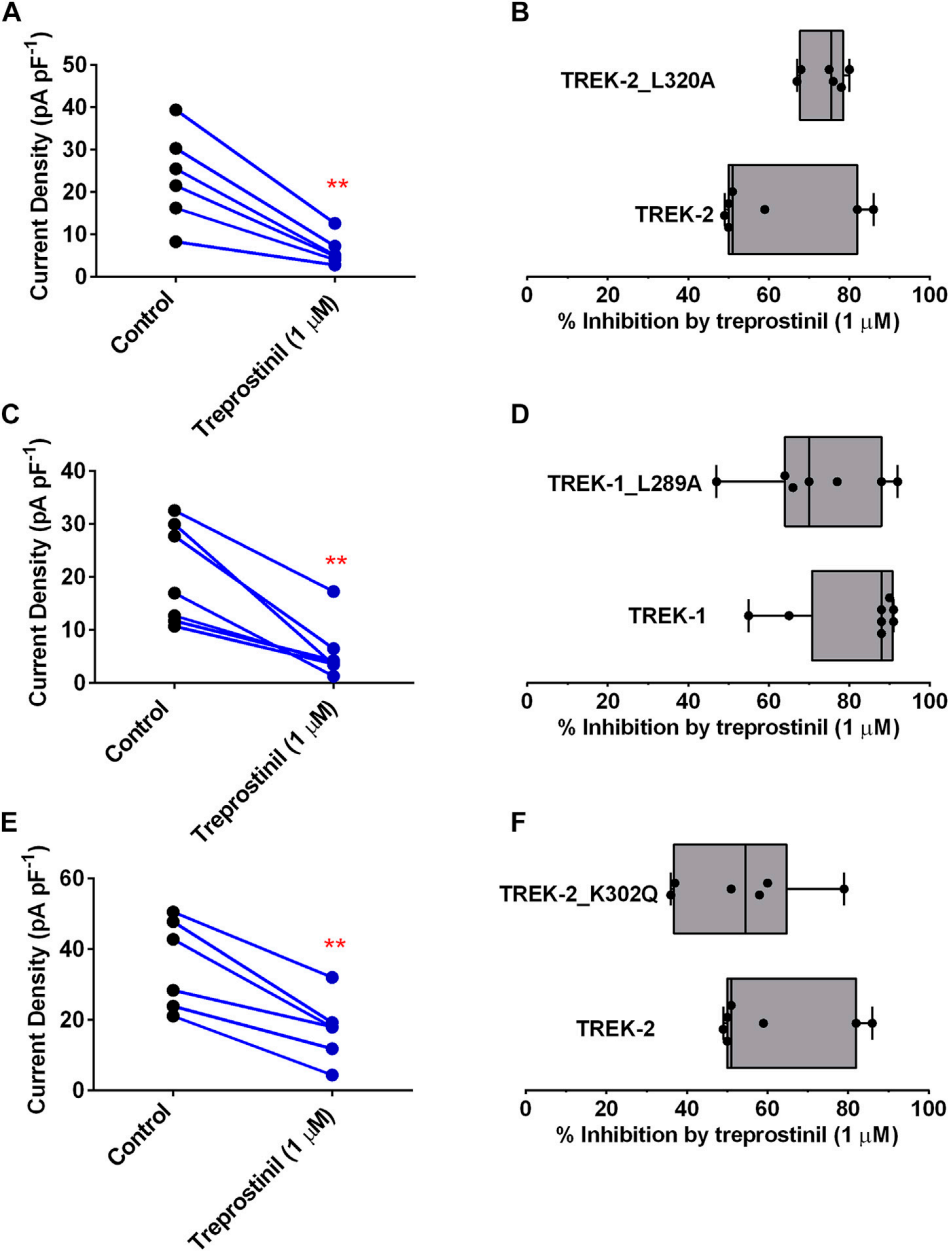
Effect of treprostinil on mutated TREK-2/L320A, TREK-1/L289A and TREK-2/K302Q (A) Measurement of whole-cell TREK-2/L320A current (pA pF^−1^) in control (*n* = 6) and following application of treprostinil (1 μM, *n* = 6, **p = 0.003; paired *t*-test) **(B)** Comparison of the inhibition of WT TREK-2 and TREK-2/L320A current by treprostinil (1 µM) calculated as the difference of current measured in control, with that measured after exposure to treprostinil, expressed as a percentage, displayed as a Box and Whiskers plot. Bars represent the min and max inhibition and lines the median inhibition, for each channel type. Points represent the individual data points **(C)** Measurement of whole-cell TREK-1/L289A current (pA pF^−1^) in control (*n* = 7) and following application of treprostinil (1 μM, n = 7, **p = 0.002; paired *t*-test) **(D)** Comparison of the inhibition of WT TREK-1 and TREK-1/L289A current by treprostinil (1 µM), expressed as a percentage, displayed as a Box and Whiskers plot **(E)** Measurement of whole-cell TREK-2/K302Q current (pA pF^−1^) in control (*n* = 6) and following application of treprostinil (1 μM, *n* = 7, **p < 0.001; paired *t*-test) **(F)** Comparison of the inhibition of WT TREK-2 and TREK-2/K302Q current by treprostinil (1 µM), expressed as a percentage, displayed as a Box and Whiskers plot.

To assess this, we mutated L320 on TREK-2 and the corresponding amino acid on the TM4 of TREK-1, L289, to an alanine. Interestingly, treprostinil-mediated inhibition of both TREK-2/L320A and TREK-1/L289A mutated channels was unchanged. The expressed TREK-2/L320A mutated homodimeric channels gave functional whole cell currents of 27.2 pA pF^−1^ (95% CI: 21.4 to 33.0, *n* = 13) that were smaller (*p* < 0.05, unpaired *t*-test) in size than WT channels 39.9 pA pF^−1^ (95% CI: 24.6 to 55.3, *n* = 7) under similar experimental conditions. Application of 1 µM treprostinil significantly reduced (*p* < 0.05, paired *t*-test) TREK-2/L320A channel currents from 23.5 pA pF^−1^ (95% CI: 12.1 to 34.9, *n* = 6) to 6.0 pA pF^−1^ (95% CI: 2.4 to 9.7, *n* = 6) (Figure 4A). Overall, the average inhibition of the TREK-2/L320A current by 1 µM treprostinil was 74% (95% CI: 68.4 to 80.0, *n* = 6), compared to 61% (95% CI: 46.1 to 75.9, *n* = 7) for WT TREK-2 channels (Figure 4B).

Similarly, the expressed TREK-1/L289A mutated homodimeric channels gave average whole cell currents of 20.3 pA pF^−1^ (95% CI: 11.6 to 29.05, *n* = 7) that were similar (*p* > 0.05, unpaired *t*-test) in size to WT TREK-1 channels and which were significantly reduced in size (*p* < 0.05, paired *t*-test) by application of 1 µM treprostinil to 5.7 pA pF^−1^ (95% CI: 0.82 to 10.64, *n* = 7) (Figure 4C). Overall, the average inhibition of the TREK-1/L289A current by 1 µM treprostinil was 72% (95% CI: 57.8 to 86.2, *n* = 7), similar to that inhibition observed for WT TREK-1 channels 82% (95% CI: 70.4 to 93.6, *n* = 8, *p* > 0.05, unpaired *t*-test) (Figure 4D).

Likewise, disruption of the cryptic modulator pocket identified in TREK, which has been shown to bind the activators ML335 and ML402, regulating the C-type gate (Lolicato et al., 2017) did not reduce the effectiveness of treprostinil on TREK-2. Mutation of the lysine (K) 302 in the TM4 of TREK-2 which sits adjacent to the selectivity filter of the channel, to a glutamine (K302Q), gave whole-cell currents of 35.7 pA pF^−1^ (95% CI: 22.2 to 49.1, *n* = 6) that were significantly reduced (*p* < 0.05) to 17.19 pA pF^−1^ (95% CI: 7.6 to 26.8, *n* = 6) in the presence of 1 µM treprostinil (Figure 4E). Overall average channel inhibition was 53.5% (95% CI: 36.6 to 70.4, *n* = 6), which was not significantly different (*p* > 0.05, unpaired *t*-test) from WT TREK-2 inhibition (Figure 4F).

A recent paper by Qiu et al. (2020), identified Y270 on TREK-1 (Y285 in their human TREK-1 construct, NM_001017425.3), as an important amino acid regulating the effectiveness of the anti-nociceptive activator, C3001a. In our hands, TREK-1 channels with the mutated amino acid Y270A carried negligible current, 3.3 pA pF^−1^ (95% CI: 2.1 to 4.5, *n* = 6) in the absence of any activator (Supplementary Figure S1), so it was not possible to determine the effect of this mutation on inhibition by treprostinil.

### A Gain-of-Function Mutation, Y284A in TREK-1, Attenuates the Inhibitory Effect of Treprostinil

**FIGURE 5:**
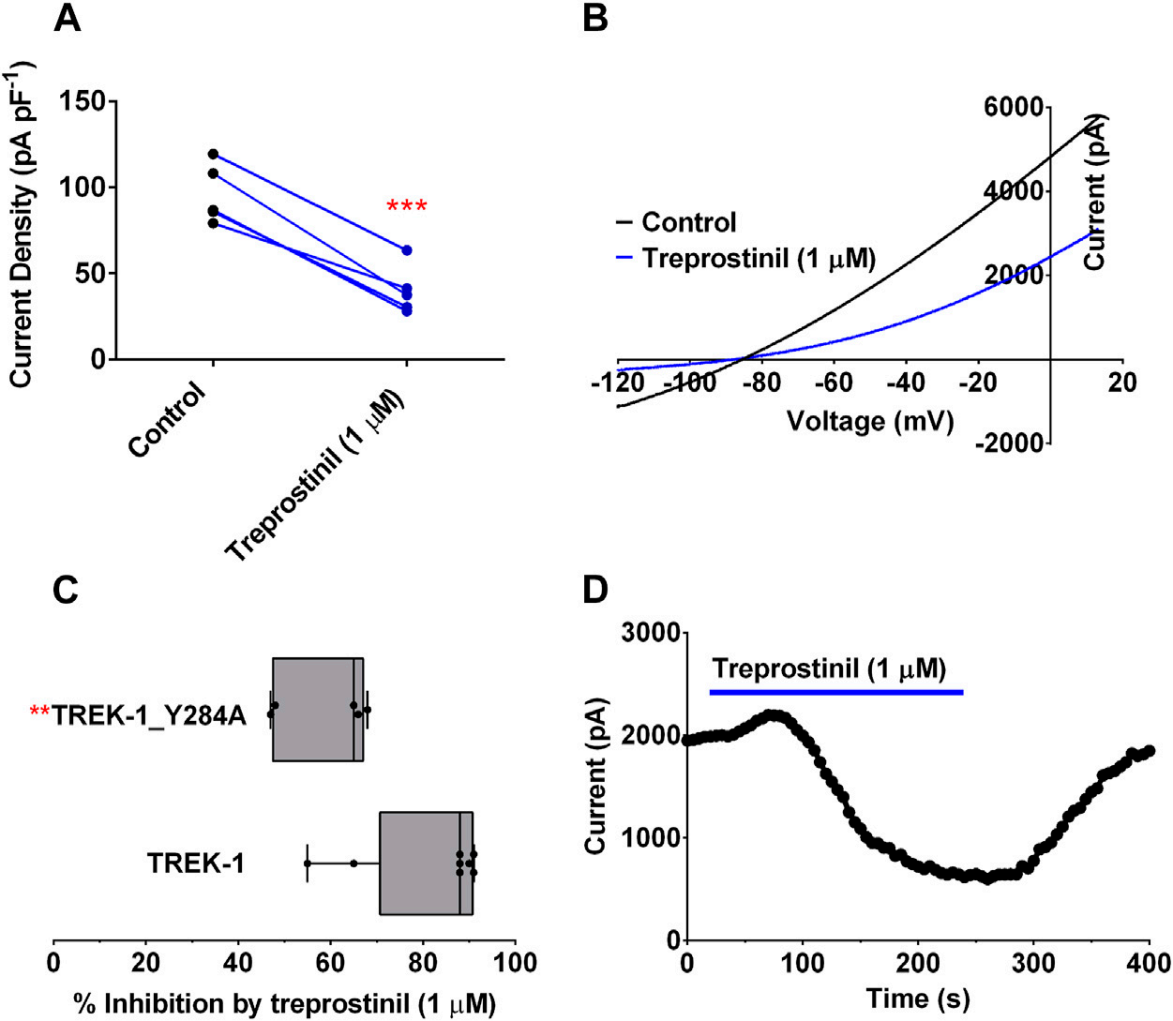
Effect of treprostinil on mutated TREK-1/Y284A **(A)** Measurement of whole-cell TREK-1/Y284A current (pA pF^−1^) in control (*n* = 5) and following acute application of treprostinil (1 μM, *n* = 5, ***p = 0.0004; paired *t*-test) **(B)** Current-voltage relationship of TREK-1/Y284A currents (average of *n* = 5 cells) in control conditions (black line) and treprostinil (1 μM, average of *n* = 5 cells, blue line) recorded over a voltage ramp (−120 mV to +20 mV) **(C)** Comparison of the inhibition of WT TREK-1 and TREK-1/Y284A current by treprostinil (1 µM) calculated as the difference of current measured in control, with that measured after exposure to treprostinil, expressed as a percentage, displayed as a Box and Whiskers plot (**p < 0.009, unpaired *t*-test) **(D)** Exemplar time course plot for TREK-1/Y284A current demonstrating the effect of changing from an extracellular control solution to one containing treprostinil (1 μM, blue line).

To try and elucidate further how treprostinil might be conferring its inhibitory effects on the TREK channels, we considered a known gain-of-function (GOF) mutation that affects the gating of the channel (Y284A). This amino acid has previously been shown to attenuate the effect of the activator, flufenamic acid (FFA) and the effect of arachidonic acid (Veale et al., 2014; Ma and Lewis, 2020) and antagonize the inhibitory effects of norfluoxetine and spadin (McClenaghan et al., 2016; Ma and Lewis, 2020). As previously reported (Veale et al., 2014) mutation of Y284 to an alanine in TREK-1, resulted in channels with large outward currents of 95.8 pA pF^−1^ (95% CI: 74.6 to 117.0, *n* = 5) which were significantly larger (*p* < 0.05, unpaired *t*-test) than WT TREK-1, 24.2 pA pF^−1^ (95% CI: 18.3 to 30.1, *n* = 8) and in agreement with channels having a higher P_o_ (Proks et al., 2020). Application of 1 µM treprostinil was found to still significantly reduce (*p* < 0.05, paired *t*-test) TREK-1/Y284A channel currents to 40.0 pA pF^−1^ (95% CI: 22.5 to 57.5, *n* = 5) (Figures 5A,B,D). Although, current through the TREK-1/Y284A mutant channel was still substantially inhibited (58%) by treprostinil, the degree of inhibition was significantly reduced (*p* < 0.05, unpaired *t*-test) from WT TREK-1 inhibition (Figures 5C,D), suggesting that the effect of the compound is influenced by gating at the channel selectivity filter (McClenaghan et al., 2016; Proks et al., 2020).

### BL-1249, an Activator of TREK Current, Antagonizes Treprostinil Inhibition of TREK-1 and TREK-2 Current

**FIGURE 6:**
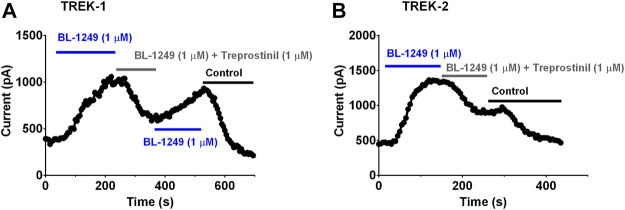
Treprostinil inhibition of TREK-1 and TREK-2 channels pre-activated by BL-1249 **(A)** Exemplar time course plot demonstrating the effect of 1 µM BL-1249 (application shown by the blue line), followed by the combined application of 1 µM BL-1249 and 1 µM treprostinil (gray line) then returning to 1 µM BL-1249 alone, on TREK-1 whole cell current **(B)** Exemplar time course plot demonstrating the effect of 1 µM BL-1249 (blue line), followed by the combined application of 1 µM BL-1249 and 1 µM treprostinil (gray line) on TREK-2 whole cell current.

The negatively charged activator, BL-1249 is an established activator of TREK-1 and TREK-2 channels (Tertyshnikova et al., 2005; Veale et al., 2014; Pope et al., 2018; Schewe et al., 2019; Mathie et al., 2021b). BL-1249 (1 µM) significantly enhanced (*p* < 0.05) TREK-1 and TREK-2 control current by 144% (95% CI: 72.8 to 214.5, *n* = 8) and 192% (95% CI: 135.2 to 248, *n* = 5) (see also Table 1, C2, D2; Figures 6A,B, blue line). Once, BL-1249-activated TREK currents were stable (2–4 min) the solution was switched to one containing both BL-1249 (1 µM) and treprostinil (1 µM) (Figures 6A,B gray line). The amount of BL-1249-activated current remaining after treprostinil inhibition, was measured once maximum inhibition by 1 µM treprostinil was observed (Table 1, C3, D3). Interestingly, for both TREK-1 and TREK-2, the current in the combined presence of treprostinil and BL-1249 (C3, D3) was not significantly different (*p* > 0.05) from the original starting current (C1, D1).

**TABLE 1:** Effect of treprostinil inhibition on BL-1249 preactivated TREK-1 and TREK-2 channels**.** Measurement of whole-cell TREK-1 current (pA pF^−1^) in control (A1) and then following application of 1 µM treprostinil (A3) (***p < 0.0004 [95% CI: −31.4 to −14.5], paired *t*-test). Measurement of TREK-2 current in control (B1) and then following application of 1 µM treprostinil (B3) (**p < 0.001 [95% CI: −34.4 to −14.1], paired *t*-test). Measurement of whole-cell TREK-1 current in control (C1) and following application of 1 µM BL-1249 (C2) (**p < 0.002 [95% CI: −69.6 to −17.10], one-way ANOVA, followed by Dunnett’s multiple comparisons test) and then following application of 1 µM treprostinil to the BL-1249-activated current (C3) (*p* > 0.05 [−31.9 to 11.76] one-way ANOVA, followed by Dunnett’s multiple comparisons test). TREK-2 current in control (D1) and following application of 1 µM BL-1249 (D2) (**p < 0.003 [95% CI: −69.6 to −17.1], one-way ANOVA, followed by Dunnett’s multiple comparisons test) and then following application of 1 µM treprostinil to the BL-1249-activated TREK-2 current (D3) (*p* > 0.05 [−38.8 to 13.66], one-way ANOVA, followed by Dunnett’s multiple comparisons test).

		1	2	3
		Current density in control	—	Current density in treprostinil (1 µM)
**A**	TREK-1	28.2 pA pF^−1^ (95% CI: 18.8–37.6) *n* = 8	―	***5.3 pA pF^−1^ (95% CI: 1.4–9.2) *n* = 8
**B**	TREK-2	39.9 pA pF^−1^ (95% CI: 24.6–55.3) *n* = 7	―	**15.7 pA pF^−1^ (95% CI: 7.3–24.0) *n* = 7
		**Current density in control**	**Current density in BL-1249 (1 µM)**	**Current density in BL-1249 (1 µM) + treprostinil (1 µM)**
**C**	TREK-1	25.3 pA pF^−1^ (95% CI: 18.6–31.9) *n* = 8	**56.9 pA pF^−1^ (95% CI: 45.4–68.3) *n* = 8	35.3 pA pF^−1^ (95% CI: 14.1–56.6) *n* = 8
**D**	TREK-2	22.9 pA pF^−1^ (95% CI: 14.5–31.3) *n* = 5	**66.2 pA pF^−1^ (95% CI: 39.6–92.7) *n* = 5	35.4 pA pF^−1^ (95% CI: 16.8–53.9) *n* = 5

## Discussion

Subcutaneous infusion of treprostinil in patients with PAH, confers significant improvements in their exercise capacity and hemodynamics, however these benefits are compromised by pain experienced at the subcutaneous infusion site (Simonneau et al., 2002; McLaughlin et al., 2003; Picken et al., 2019). Treprostinil treatment has also been shown to have several other painful side effects, including jaw pain, headache, and pain in extremities (Picken et al., 2019). In this study we have shown that treprostinil potently and reversibly inhibits, both human TREK-1 and TREK-2 channels. TREK-1 and TREK-2 channels are strongly implicated in pain signaling pathways and both are expressed abundantly within sensory neurons (Alloui et al., 2006; Marsh et al., 2012).

In contrast, TASK-1 channels were not inhibited by treprostinil. TASK-1 channels are highly expressed in human PASMCs and have a role in regulating the resting membrane potential of these cells (Olschewski et al., 2006). Downregulation, inhibition or inactivating mutations of TASK-1 channels have been shown to contribute to detrimental vascular remodeling in the PAH disease phenotype (Ma et al., 2013; Boucherat et al., 2015; Antigny et al., 2016; Navas et al., 2017; Cunningham et al., 2019), while pharmacological interventions that enhances TASK-1 channel current have been found to be beneficial in PAH treatment (Olschewski et al., 2006; Ma et al., 2013; Antigny et al., 2016; Cunningham et al., 2019). Treprostinil has previously been demonstrated to enhance TASK-1 channel current indirectly via a protein kinase A-dependent pathway (Olschewski et al., 2006). This is in agreement with the data obtained here, where prolonged incubation in treprostinil was found to induce an enhancement of current through TASK-1 channels.

### Regulation of TREK Channels by Treprostinil

A distinguishing feature of K2P channels is the existence of an extracellular cap domain, which causes the formation of two tunnel-like side portals permitting bilateral extracellular ion conduction from the selectivity filter (Brohawn et al., 2013; Dong et al., 2015). This structural feature underlies the insensitivity of K2P channels to classical K channel pore blockers such as TEA (Brohawn et al., 2012; Miller and Long, 2012). Some small molecules, such as ruthenium red and zinc exert their inhibitory effect through sites on the extracellular cap of TASK channels (Czirják and Enyedi, 2003; Musset et al., 2006; Clarke et al., 2008). More recently, the extracellular cap in TREK channels has been demonstrated to be important in transducing the effect of small ligands, such as TKDC and ruthenium red, via allosteric conformational changes of the bilateral side portals that subsequently interrupts ion conduction (Braun et al., 2015; Luo et al., 2017). In order to elucidate the molecular mechanism by which treprostinil inhibits TREK channels, we studied its effect on three identified mutations in the extracellular cap, which have been shown to be important for TKDC binding to TREK-1 channels (Luo et al., 2017). Surprisingly, none of these mutations altered the inhibitory effect of treprostinil on TREK-1 channels, suggesting that treprostinil does not exert its effect by binding to this region of the extracellular cap and blocking the ion conduction pathway of the channel.

Another TREK channel antagonist and antidepressant agent, norfluoxetine (Kennard et al., 2005), exerts it effects through binding to a highly conserved fenestration adjacent to the pore filter entrance of TREK-2 (Dong et al., 2015). Contact points for norfluoxetine on TREK-2 were identified in the TM4 region of the channel and mutation of L320 was found to reduce norfluoxetine inhibition of TREK-2 (Dong et al., 2015). The equivalent amino acid on the TM4 of TREK-1, L289 has also been identified as a contact point for the TREK-1 agonist, BL-1249, mutation of which reduced the effectiveness of the compound (Schewe et al., 2019). We determined whether treprostinil was exerting its inhibitory effect via the equivalent amino acids on the intramembrane fenestrations of both TREK-1 and TREK-2, however, mutations of antagonist sites in the TM4 region of both TREK-1 and TREK-2, did not alter treprostinil inhibition of the channels. This would imply that treprostinil does not bind to the same site on TREK channels as norfluoxetine.

Lolicato et al. (2017) showed that two small molecule activators of TREK channels, ML335 and ML402, were able to influence the C-type gate independently of the TM4 region of the channel, by binding directly to the C-type gate, coined the “cryptic selectivity-filter binding site” (Lolicato et al., 2017). A lysine in TREK-1 (K271) was identified as being an important interaction site for these compounds and essential for activation (Lolicato et al., 2017). Mutation of the equivalent lysine in TREK-2 (K302Q) did not alter the effect of treprostinil.

A tyrosine in the TM4 region (Y270 in the TREK-1 isoform used here) was recently shown to be important in regulating the effectiveness of an anti-nociceptor activator, C3001a (Qiu et al., 2020). We found, however, that this mutation carried negligible current at −40 mV in the absence of a high concentration of a TREK-1 channel activator and thus were unable to test whether it interfered with treprostinil inhibition.

### Attenuation of Treprostinil Inhibition of TREK Channels

The C-type selectivity filter gate is highly sensitive to intracellular modulators such as heat, mechanical stress and intracellular acidosis (Patel et al., 1998.; Maingret et al., 1999; Bagriantsev et al., 2011; Piechotta et al., 2011; Mathie et al., 2021b) and extracellular regulators such as protonation (Cohen et al., 2008; Sandoz et al., 2009). A number of amino acids in the pore lining TM4 helix of TREK-1 close to the selectivity filter have been identified as important for the regulation of channel gating. Mutation of these amino acids give rise to a GOF that in turn interferes with channel gating and regulation by both external and internal regulators (Bagriantsev et al., 2011; Bagriantsev et al., 2012; Veale et al., 2014). We have previously shown that a tyrosine at position 284 in TREK-1 causes a GOF that substantially reduces activation by the fenamate, flufenamic acid and by BL-1249, reducing ligand efficacy (Veale et al., 2014). This tyrosine residue, located within the “hinge” of the TM4 domain of TREK channels, a critical area involved in the movement of these channels between different “up” and “down” states (Dong et al., 2015), has been shown to alter the channels sensitivity to stretch, norfluoxetine and BL-1249 (McClenaghan et al., 2016; Aryal et al., 2017; Schewe et al., 2019). Mutation of this amino acid in TREK-2 (Y315A, Dong et al., 2015) pushes the channel into an activated state, with increased macroscopic currents and higher resting open probability (P_o_) (McClenaghan et al., 2016; Proks et al., 2020). This region has also been shown to be important in the binding of both channel activators and inhibitors in other K channels (Hosaka et al., 2007; Perry et al., 2010; Garg et al., 2011). Interestingly, we found that the equivalent, Y284A, GOF mutation of TREK-1, significantly attenuated the effect of treprostinil on these channels, suggesting that treprostinil was much less efficient at inhibiting these channels when it was gated open (McClenaghan et al., 2016; Proks et al., 2020). Thus, although treprostinil does not bind to the same site as norfluoxetine, block by treprostinil is also gating-state dependent and is more effective when TREK channels are in their lower open probability state, characterized by the TM4 regions of the channels being in their “down” conformation (Dong et al., 2015; McClenaghan et al., 2016). As such, the effect of treprostinil is influenced by “C-type” selectivity filter gating of the channel (Chemin et al., 2005; Bagriantsev et al., 2011, Bagriantsev et al., 2012; Dong et al., 2015; McClenaghan et al., 2016). It is of interest that inhibition by treprostinil of TREK-1 (∼75%) and TREK-2 (∼55%) is incomplete which is consistent with the presence of multiple gating states of TREK channels (McClenaghan et al., 2016), one or more of which is resistant to block by the compound.

Since gating of the channel influenced treprostinil inhibition, we determined whether gating the channel “open” using a pharmacological activator, BL-1249 (Tertyshnikova et al., 2005; Veale et al., 2014) would also attenuate the inhibitory effect of treprostinil. We found that pre-application of BL-1249 to both TREK-1 and TREK-2 channels did indeed attenuate the inhibition by treprostinil, as we saw with the GOF mutation. Indeed, the treprostinil inhibited current was greater than the original baseline starting current, pre-BL-1249 treatment. BL-1249 pre-activation of TREK-1 current has been shown to antagonize the effect of the inhibitor, spadin (Ma and Lewis, 2020), a similar effect to that observed for TREK-2 current with the activator ML335 and inhibitor norfluoxetine (Lolicato et al., 2017; Proks et al., 2020).

Given that TREK channels are known to participate in acute nociception it is possible that the inhibition of TREK channels by treprostinil is responsible for the subcutaneous site pain experienced by PAH patients. This is similar to the acute pain induced by oxaliplatin when used in cancer therapy (Poupon et al., 2018) which is attributable to a down regulation of TREK and TRAAK channels (Pereira et al., 2021). The mean plasma concentration of treprostinil in patients is around 10–20 ng/ml (25–50 nM) which is in the range of the IC_50_s seen here for block of TREK-1 and TREK-2 channels. At the site of subcutaneous injection however, this concentration would be higher and able to cause substantive block of TREK channels. The potent antinociceptive benefits of pharmacologically upregulating TREK-1 and TREK-2 channels has recently been shown *in vivo* with a series of substituted acrylic acid analogues and C3100a (Vivier et al., 2017; Qiu et al., 2020). Similarly, activation of TREK channels by riluzole can overcome the neuropathic pain induced by oxaliplatin (Poupon et al., 2018). It is proposed that the use of a specific activator of TREK channels, applied topically, could potentially provide a safe therapeutic strategy to overcome the excruciating pain that is experienced by patients receiving treprostinil injection.

## Data Availability

The raw data supporting the conclusions of this article will be made available by the authors, without undue reservation.
