# Supplementary material for: The Prostacyclin Analogue, Treprostinil, Used in the Treatment of Pulmonary Arterial Hypertension, is a Potent Antagonist of TREK-1 and TREK-2 Potassium Channels
Source: Front Pharmacol. 2021 Jun 29;12:705421. doi: 10.3389/fphar.2021.705421 (PMC8276018; doi:10.3389/fphar.2021.705421)
Supplement: Supplementary file 1 [file DataSheet1.pdf]

Figure S1: Mutation of tyrosine 270 in TREK-1 causes a reduction in basal current.

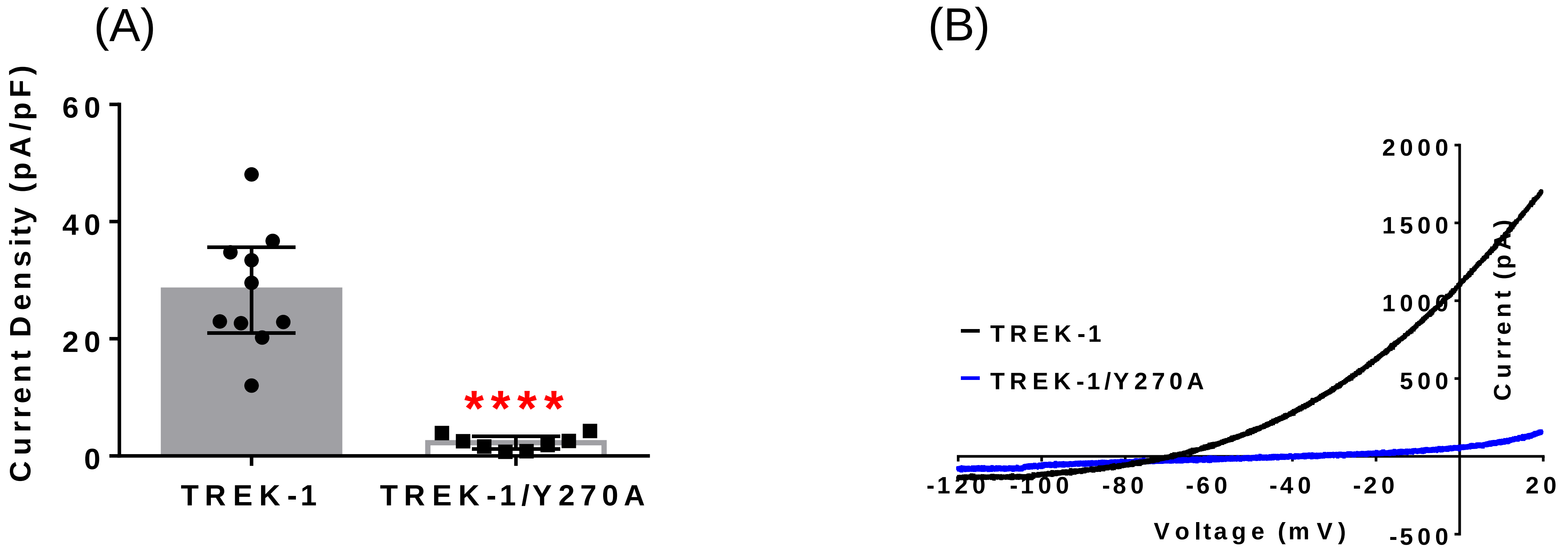

Figure S1: Mutation of Y270 on TREK-1 alters channel function. (A) Bar graph of current densities (pA pF^{-1}) from tsA201 cells transiently expressing WT TREK-1 (n = 10) and TREK-1/Y270A (n = 8) mutated channels. Error bars represent the 95% CI and symbols represent currents measured from individual cells. ****$p < 0.0001$ [95% CI: -33.8 to -18.2], unpaired t-test. (B) Current-voltage plot of WT TREK-1 (black line) and TREK-1/Y270A (blue line) currents recorded over a voltage ramp (-120 mV to +20 mV).
